# Investing in the Prevention of Communicable Disease Outbreaks: Fiscal Health Modelling—The Tool of Choice for Assessing Public Finance Sustainability

**DOI:** 10.3390/vaccines11040823

**Published:** 2023-04-10

**Authors:** Simon van der Schans, Marcel H. Schöttler, Jurjen van der Schans, Mark P. Connolly, Maarten J. Postma, Cornelis Boersma

**Affiliations:** 1 Health-Ecore B.V., 3700 AA Zeist, The Netherlands; 2 Unit of Global Health, Department of Health Sciences, University Medical Center Groningen (UMCG), University of Groningen, 9713 GZ Groningen, The Netherlands; 3 Department of Economics, Econometrics and Finance, Faculty of Economics & Business, University of Groningen, 9747 AJ Groningen, The Netherlands; 4 Department of Management Sciences, Open University, 6419 AT Heerlen, The Netherlands; 5 Unit of PharmacoTherapy, Epidemiology and Economics, Department of Pharmacy, University of Groningen, 9713 AV Groningen, The Netherlands; 6 Global Market Access Solutions, 1162 Saint-Prex, Switzerland

**Keywords:** fiscal health modelling, governmental perspective, tax revenue, social benefits, infectious disease, COVID-19

## Abstract

National strategies for preparedness for future outbreaks of COVID-19 often include timely preparedness with vaccines. Fiscal health modelling (FHM) has recently been brought forward as an additional analysis by defining the public economic impact from a governmental perspective. As governments are the main decision-makers concerning pandemic preparedness, this study aimed to develop an FHM framework for infectious diseases in the Netherlands. Based on the Dutch COVID-19 outbreak of 2020 and 2021 and publicly available data on tax income and gross domestic product (GDP), the fiscal impact of COVID-19 was assessed using two approaches. Approach I: Prospective modelling of future fiscal impact based on publicly available laboratory-confirmed COVID-19 cases; and Approach II: Retrospective assessment of the extrapolated tax and benefit income and GDP. Approach I estimated the consequences that can be causally linked to the population counts reducing income taxes by EUR 266 million. The total fiscal loss amounted to EUR 164 million over 2 years (excluding pension payments averted). The total losses in terms of tax income (2020 and 2021) and GDP (2020) (Approach II), were estimated at, respectively, EUR 13.58 billion and EUR 96.3 billion. This study analysed different aspects of a communicable disease outbreak and its influence on government public accounts. The choice of the two presented approaches depends on the perspective of the analysis, the time horizon of the analysis and the availability of data.

## 1. Introduction

The outbreak of a communicable disease (CD) can have large consequences on the general health and daily life of societies. The global SARS-CoV-2 (COVID-19) pandemic has been an extreme example of this. At the pandemic’s peak, societies and governments struggled to cope with large infectious waves, placing strains on national healthcare systems and public finances [1]. Aiming to reduce the spread of the virus, governments implemented a multitude of non-pharmaceutical interventions to prevent the spread of infections and to reduce hospitalizations and deaths. However, these interventions brought forward consequences in other economic domains which were not necessarily captured in the epidemiological models used to steer these interventive policies in the first place [2,3,4].

Given the extent of the impact of the COVID-19 pandemic and counteracting measures—in lives lost, reduced population health and economically—the necessity to examine CD-counteracting healthcare interventions with a broad perspective, reaching beyond the current health technology assessment (HTA) practice, is highlighted. Even additional scenario analyses, such as future unrelated healthcare costs which sometimes are added to conventional cost-effectiveness analyses in HTA procedures, do not fully capture the broader economic consequences of a CD outbreak such as COVID-19. Governments around the world have now stressed the importance of preparedness for future CD outbreaks through the means of the timely availability and executability of vaccination campaigns requiring upfront investment. Consequently, this will create the need for public decision-makers to assess different vaccines and (emergency) policies for their respective benefits and costs, incorporating the broad perspective described above [5]. The pandemic has exposed the need for a currently lacking, structured approach to assess the broad impact of interventions, both pharmacological and non-pharmacological, in response to CDs.

In the past, vaccination campaigns have been investigated regarding the added health benefit relative to the added costs for society (cost-benefit analysis). However, in the field of health economics, fiscal health modelling (FHM) has recently been brought forward as an estimation technique specifically focused on quantifying the consequences of health policies from a public finance perspective [6]. Multiple publications have previously elaborated on this approach, particularly in the context of vaccination [7,8]. It was suggested that FHM is useful as a complementary analysis for budgetary decision-makers, due to its approach to presenting a clear investment case, compared to the frequently used cost-effectiveness assessments [8]. However, when it comes to communicating the financial benefit of reducing the broad impact of a future CD outbreak through vaccination, there is a lack of definition of an FHM analysis scope, efficiently summarizing the number of societal and health areas affected.

This study aims to determine such scope by defining an FHM analytic framework for vaccination strategies as part of societal and governmental pandemic preparedness policies. Secondly, using the real-world example case of COVID-19 in the Netherlands, we aim to retrospectively quantify the fiscal impact of the COVID-19 pandemic on the Dutch government. The goal of this manuscript is to develop a fiscal health modelling framework for the Netherlands to calculate the fiscal impact of CD based on previously published literature.

## 2. Materials and Methods

### 2.1. Fiscal Health Modelling

FHM estimates the effects of a health condition on governmental net lifetime tax value and average government transfer payments following changes in morbidity and mortality rates due to a disease and/or intervention. As a result of the significant cross-sectoral impact of a CD and the public urgency of an outbreak, interventions aiming to counteract often fall under the responsibility of national governments. Yet, these might be outside the funding responsibilities of national health authorities generally covering health care. Hence, FHM is suited to policymakers and budgetary holders outside the health sector that requires a different set of economic data to inform policy.

### 2.2. Research Design

Health economics is characterized by two perspectives that are often used to inform decision-makers regarding budget allocation in health care. The payer and societal perspectives are most commonly used. In the Netherlands, the societal perspective is the primary perspective as prescribed by the Dutch guidelines for economic evaluations [9]. In the case of public health, and CDs especially, these two perspectives might not be sufficient. Governments are the primary payer of care in public health interventions and prevention of or response to CD outbreaks. Applying the standard societal perspective would include costs that are less relevant and do not include effects that directly influence governmental accounts. Publicly funded health interventions and health systems would benefit from an economics assessment from the perspective of the primary payer. The governmental perspective has been brought forward as an additional perspective to the payer and societal perspectives. In the Netherlands, there are no clear guidelines or methodologies to assess the fiscal impact of CDs and/or health interventions. In 2018, Mauskopf et al. published a paper regarding the economic evaluation of vaccination programs [6]. This paper discussed and recommended different types of economic analysis relevant to CDs. Cost-effectiveness analysis, constraint optimization modelling and fiscal health modelling were presented as economic analysis approaches relevant to the assessment of vaccines and infectious diseases. Fiscal health modelling is described as an approach to estimate the net present value of government revenues and expenditures attributed to disease effects.

This study was performed in the context of previously published literature regarding FHM, the governmental perspective on health technology assessment (HTA) and the impact of COVID-19 in the Netherlands. The COVID-19 pandemic has highlighted the importance of a structured and improved assessment of responses to CDs from the governmental perspective. These methods, while previously described, have not yet been quantified for the Netherlands and could substantially impact and improve the decision-making regarding CDs in the Netherlands. A scoping literature review from the core literature identified and supplemented with internal expert opinion meetings regarding methodology and fiscal categories was performed.

### 2.3. Data Collection

The fiscal health modelling framework was developed based on previously published available literature. The studies by Mauskopf et al., Connolly et al. and Kotsopoules et al. provided the basis for the fiscal health modelling framework and were expanded through a scoping literature search from the previously mentioned relevant papers [6,7,10,11,12,13,14]. The relevant fiscal categories were identified from the literature and quantified through a literature search regarding the Dutch tax and social benefits rules and regulations; an overview per category is shown below [15,16,17,18,19,20,21,22,23,24,25,26]. Identification and inclusion of fiscal categories and/or exclusion of fiscal categories were discussed by two authors (SvdS and MS). In the case of disagreement, the case was discussed with the other authors until consensus was reached. The fiscal categories were identified through an assessment of the Dutch tax and social benefit system, discussed within the team of authors of this manuscript and supplemented with an expert opinion review with experience in fiscal health modelling. Based on these results, we analysed the Dutch governmental websites regarding tax and social benefits relevant to the fiscal categories shown in Table 1. The primary fiscal categories identified are income tax, value added taxes (VAT), inheritance tax, social benefits and state pension payments.

### 2.4. Framework

**Figure 1:**
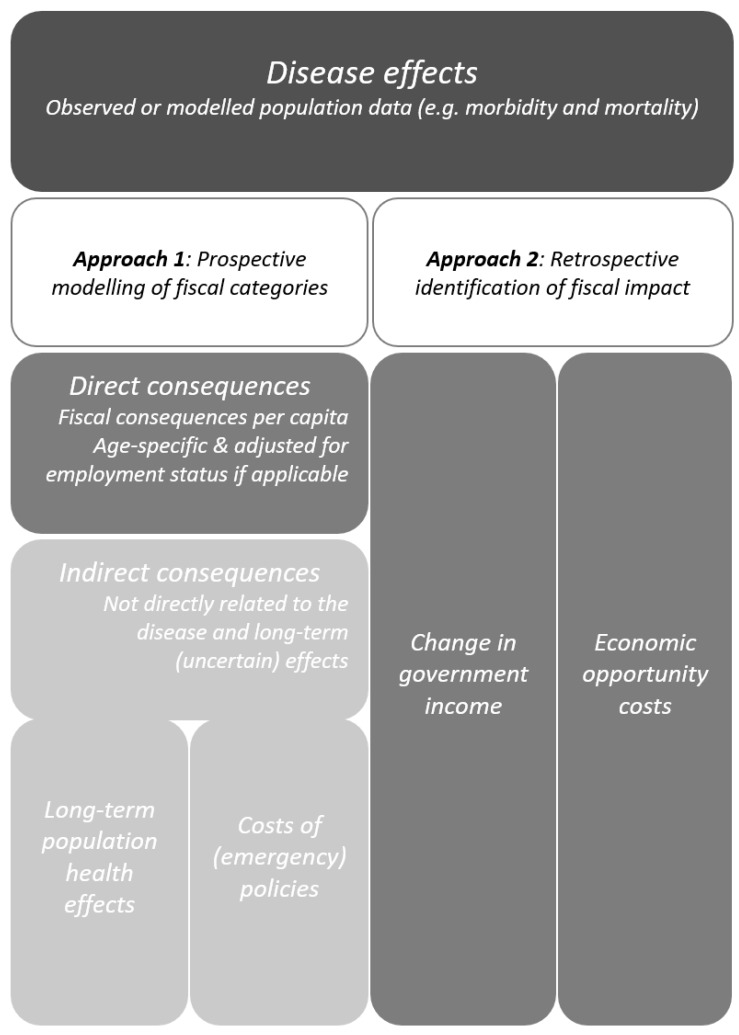
Framework schematic overview. The disease effects (population data) of the first analysis approach are depicted on the left of the figure. The alternative approach is shown on the right.

Based on the literature search and expert opinions, we identified two possible approaches to estimate the fiscal impact of CD. Under the first approach, direct consequences which can be causally linked to an infectious case should be determined and summed up. This procedure is followed by more uncertain or indirect consequences, which should then be estimated. The second approach to estimating the fiscal impact we propose is to use simple regression analysis to compare an estimated change in fiscal categories with the realized value observed in 2021. A visual representation of the proposed framework is depicted in Figure 1. In the next sections, we detail the different approaches.

For the first approach to fiscally analysing a CD outbreak, the absolute amount of disease effects should be collected in the form of population data, reporting the number of individual laboratory-confirmed registered cases of COVID-19 within a period.

#### 2.4.1. Base Inputs

Given the potentially broad health impact of a CD outbreak within society, we propose this analysis to assess disease effects which are reported with conventional population data or population data from HTA models. Data on the cases of infections, hospitalizations and mortality are expected to be the most readily available form of reliable data. Following the fiscal profiles of different age groups, count data should additionally be categorized into age cohorts. Analysts should determine the number of age cohorts by trading off/weighting the difficulty of obtaining data on smaller age cohorts as well as the consequent model complexity against the achieved incremental model accuracy.

#### 2.4.2. Population Data

Dutch *COVID-19*-specific population data on laboratory-confirmed infections, hospital admissions, intensive care unit (ICU) admissions and mortality, were collected for the pre-specified timeframe using multiple publicly available data files of the Dutch Institute of Public Health (RIVM) [34]. The population data includes all laboratory confirmed *COVID-19* cases in the Netherlands. Infection and hospitalization data were processed using R (version 4.0.3) to group individuals into five age cohorts: 0 years–19 years; 20 years–39 years; 40 years–59 years; 60 years–79 years; 80+ years [35].

### 2.5. Approach I

#### 2.5.1. Direct Consequences of the CD

The collected population data can be connected to fiscal consequences for which there has been a proven causal relationship, or which can be logically inferred. For example, a deceased person cannot pay income tax anymore, which consequently represents a loss to the government. Aiming to capture all influences of the CD outbreak, researchers should use the accounting principle of receivables and payables to assess how a health condition influences tax revenue and government payments in relation to specific health events. Preferably, fiscal values on different payables and receivables should be specific to the previously determined age cohorts to enable a more detailed analysis of the final fiscal impact. In addition, the mentioned fiscal values should be adjusted for the employment status of the included individuals [27].

To translate the disease effects collected above, the Dutch social system was the basis for the determination of the direct fiscal consequences. The fiscal inputs were recalculated to 2020. The receivables of the state in this analysis consisted of income tax on salary, value-added tax (VAT) revenue and inheritance tax revenue due to mortality. The framework allows the inclusion of payables in the form of social benefits due to newly incurred work disability.

**Figure 2:**
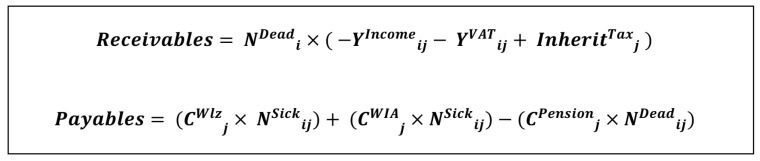
Abbreviations: N: number of subjects, Y: Tax revenue, Inherit: Inheritance, i: per age cohort, j: per capita, C: pay-out category (costs), Wlz: Long-term care, WIA: Work disability.

As described above, the FHM analysis in Approach I sums up the fiscal results per health state as a result of mortality and morbidity. The collected patient data per specified age cohort were combined with the quantified estimates of the receivables and payables using the specified equations in Figure 2. The change in receivables of the Dutch state is calculated as the product of the number of deceased individuals and the sum of lost income tax as well as VAT and the gained inheritance tax. The change in payables is the sum of long-term care, work disability benefits and averted pension payments. The mentioned categories of receivables and payables are described in more detail below.

#### 2.5.2. Receivables

To calculate the income tax per capita, the gross income per age group was collected from the Central Bureau of Statistics and tax authorities [15]. The income tax revenue per capita was calculated based on the Dutch tax brackets and tax credit [16,17,18]. The average income tax revenue was corrected for labour participation [27]. The average income tax per capita per age cohort is shown in Appendix A, Table A1. Additional tax revenue losses can occur as a result of morbidity. The data necessary to calculate this was not available, but the potential tax revenue losses per capita per year are shown in the Results. The change in VAT revenue was calculated based on a publication by van der Schors et al. on the average spendable income per household in the Netherlands [26]. In order to calculate the average VAT spending per capita, the gross income per household, the number of persons per household and the average VAT percentage were used [26,28]. There were no data specifically for the age group 0–19, and we therefore assumed that these had no VAT expenses. The average VAT revenue per capita per age cohort is shown in Appendix A, Table A2. The VAT revenue change included in the direct consequences is solely due to the direct effects of mortality. The VAT revenue due to possible, yet uncertain change in consumer behaviour is analysed under indirect consequences below. The inheritance tax was calculated based on the total bequeathed assets and the total number of deceased in 2018 [21,29]. The average inheritance tax was calculated based on the weighted average tax rate (tax rate weighted to who receives the inheritance) and exemption until a predefined amount [19,20]. The average inheritance tax was EUR 17.144 per inheritance.

#### 2.5.3. Payables

In the Dutch social security system, salaries are continued to be paid out for a minimum of 70% for up to 2 years in the case of work disability by the employer [25,31]. After 2 years, the employee falls under the national incomes insurance scheme (WIA: ’wet werk en inkomen naar arbeidsvermogen’) [23]. In the case of partial disability, which results in a reduced salary below a government-set social minimum, the individual has the right to supplementary social benefits to sustain that minimum. Alternatively, in the case of full disability, an individual will receive social benefits up to 75% of the salary earned in the year before disability [23].

Another possible payment of social benefits is the WLZ (‘wet langdurige zorg’). The WLZ is between 85% and 100% of the income of someone who requires (intensive) care and is fully unable to work [32]. There are predefined indications related to the WLZ; in most cases of CD infections, these will not occur. Social benefit payments for support for partners and family members in the case of mortality of an individual (ANW: ‘algemene nabestaandenwet’) were excluded [24,36]. As social benefits in their extent are highly dependent on the personal (financial) situation, and only in the case of mortality and the partner or children living below the social minimum, this study did not utilize pay-out for this benefit category.

In the particular case of COVID-19 in the Netherlands, no data on the utilization of long-term care and work disability benefits were yet available at the population level, which is why the WLZ and WIA have not yet been quantified. Further financial and other economic support measures were excluded from these equations, as these focus only on the epidemiological impact of the virus. The average state pension payments were calculated to be EUR 11.686 annually per capita [33]. To capture all possible effects as a result of the COVID-19 pandemic, we assumed a lifetime time horizon. Following the Dutch guidelines for social-economic cost-benefit analyses, an annual discount rate was set at 4% per year for all included fiscal inputs [9].

#### 2.5.4. Long-Term Consequences

As many implications and consequences of the COVID-19 outbreak are uncertain, this study quantifies exemplary long-term consequences, as mentioned and qualified in the framework above. An infection of a CD can also have consequences which are more long-term and uncertain in their nature than the outbreak itself. Hence, consequences not specifically quantified in the literature should be included separately from the direct consequences in the form of hypotheses or value ranges. We propose to categorize these consequences into two areas:

#### 2.5.5. Long-Term Population Health Effects

An example of a long-term change in population health is the novel incidence of self-reported long-lasting symptoms after a COVID-19 infection (Long-Covid). As an example in this category, published estimates regarding Long-Covid were sourced from a population survey by the British Office of National Statistics [37]. From this UK Long-Covid data, population estimates for the Netherlands were calculated per age group, based on the percentage of Long-Covid of the total British inhabitants. The different levels of severity were as follows: activity not limited, activity limited and activity limited severely [34,37]. The Long-Covid data used in the model are shown in Appendix A, Table A3. To quantify the possible fiscal impact of morbidity, the rates of activity impairment were applied to projected earnings in each age cohort and used to estimate tax revenue losses, i.e., receivables. Subsequently, we applied the Dutch statutory payments by impaired activity level to derive increases in receivables for each age category. These levels express an individual’s inability to work with a percentage value relative to the work capacity of an individual without a health condition. The included levels in this study were 25%, 50%, 75% and 100% inability to work.

#### 2.5.6. Costs of Emergency Policies

The cost of emergency policies are all expenses that can be identified from a governmental perspective as a response to the COVID-19 pandemic. The costs are collected from reports of the Dutch court of audit [38]. They include measures of liquidity support by the government to the corporate sector to retain mass employment and prevent bankruptcies. Furthermore, they focus on enabling the health sector to cope with the new demand. However, more indirect consequences also required funding, such as the educational sector when aiming to reduce the learning disadvantage for some pupils with weak socioeconomic backgrounds.

### 2.6. Approach II

Alternatively, fiscal categories in their entirety can be identified for the respective research setting and extrapolated. Generally, big fiscal ledgers can be considered, such as the general governmental tax or premium income, i.e., national accounts or the development of the national GDP in the pre-determined period. Following this approach, a simple regression fit should be conducted on the historical data per category to enable the (hypothetical) comparison of a situation with and without an outbreak. A type of consequence which was explicitly not included in the framework was absenteeism and presenteeism losses because this mostly impacts employers and rarely the direct financial detriment of the government.

#### 2.6.1. Change in Government Tax Income

Another possibility to express the fiscal impact of the COVID-19 pandemic is the incremental change to governmental general tax revenue when comparing the pre- and post-COVID-19 years [21]. Fiscal tax income data from the period of 2012–2019 published by CBS were fitted with an exponential function. This function was then used to estimate tax revenue for 2020 and 2021. The estimated (trend) values were sequentially compared with the actual (e.g., realised) values to calculate a change in overall governmental revenue.

#### 2.6.2. GDP Opportunity Costs

To assess the lost gross domestic product (GDP) due to the pandemic as a form of opportunity cost, reported GDP figures in the time frame of 1990–2019 were extrapolated using a linear function [39]. The Dutch GDP values for the year 2020 were converted using Purchasing Power Parity (PPP). The estimated figure for 2020 was then compared with the actual reported numbers for that year. Consequently, the difference offers a rough assessment of the GDP lost through opportunity costs by the pandemic. Up to this point, no concrete estimation for 2021 is yet published; this year was therefore excluded from this particular category.

### 2.7. Calculations: Quantifying the Fiscal Impact—A Real-World Case

As a case example, this study aimed to quantify the fiscal impact of the COVID-19 pandemic in the Netherlands between the 1 January 2020 and the 31 December 2021 following the framework described above. The FHM model was quantified using Excel 2016^®^ following the FAST financial modelling principles for financial modelling [40], a commonly used method in financial modelling. Due to the availability of fiscal data, outcomes were shown and calculated for the years 2020 and 2021 for both alternative approaches for FHM, as presented above. Because of the uncertainty around the magnitude of indirect consequences (in Approach I) two years after the start of the pandemic, we quantified examples for each of these categories.

### 2.8. Lockdown & Vaccination Data

Because, in the Netherlands, multiple forms of interventions were introduced during the above-mentioned period, these were compared to the infections, hospitalization, ICU admissions and mortality and their respective effect on receivables and payables [41]. Interventions regarding the closing of public locations, the closing of educational institutions, day and night catering industry and partial or full lockdowns were analysed as interventions [41]. The vaccination rate was calculated for single vaccinated, fully vaccinated and people that received one booster vaccination [42]. Data on these three vaccination statuses were calculated based on the Dutch inhabitants in February 2022 [43].

## 3. Results

### 3.1. Disease Effects

**Figure 3:**
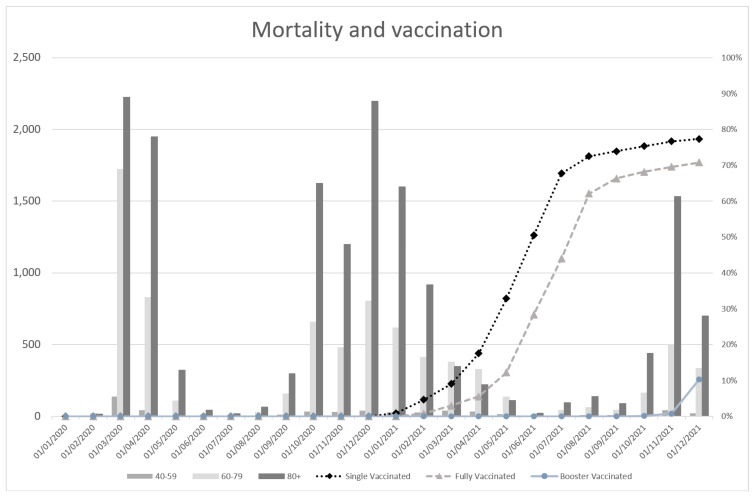
Total mortality and vaccination rate over time. The mortality in the Netherlands over time is displayed using bar graphs. The vaccination level over time relative to the overall Dutch population is shown using the line graphs.

In 2020, the total number of COVID-19 infections was 826,086, followed by 35,920 hospitalizations and 6694 ICU admissions, and the overall mortality was 15,102. In 2021, the total number of infections was 2,356,933, the number of hospitalizations was 52,678, ICU admissions happened 9805 times and mortality was 9493. Between 2020 and 2021, the number of infections, hospitalisations and ICU admissions shifted towards the lower age groups in attribution to the total in each category. No such dynamic was observed with mortality. The population per age group and category is shown in Appendix A, Table A4a,b ((a) (2020) and (b) (2021)). The total mortality over time and vaccination rate is shown below in Figure 3.

### 3.2. Approach I

#### 3.2.1. Direct Consequences

**Figure 4:**
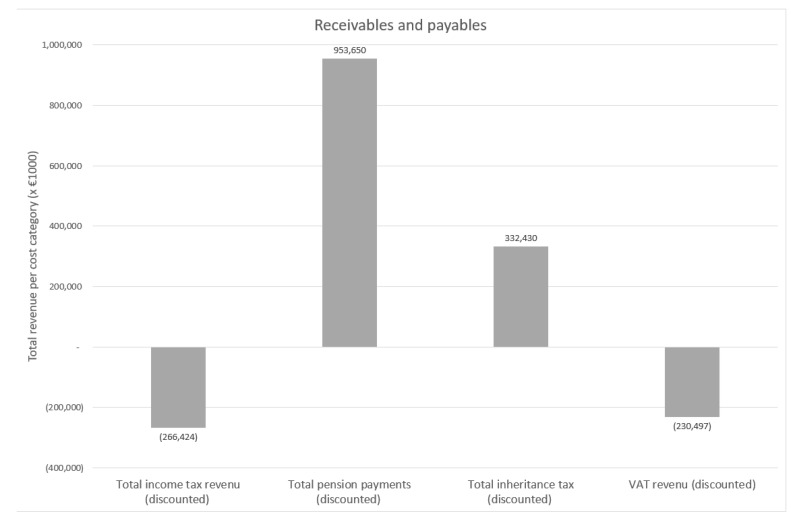
Total amount of receivables and payables. Description: The values were derived from multiplying population data in 2020 and 2021 with the age-group-specific consequences on the four government cost categories. The effects of COVID-19 on work disability were excluded, as described in more detail earlier.

Based on the population data described above and the connected direct fiscal effects per subject highlighted earlier, the total loss of income tax attributed to COVID-19 mortality was equal to EUR 266 million. The total lost VAT income was EUR 230 million, whereas the total inheritance taxes gained was estimated to be EUR 332 million. The total state pension payments averted were EUR 953 million due to mortality. This resulted in lost receivables of EUR 164 million combining both 2020 and 2021. As no direct consequences of morbidity (long-term care, work disability) were included in the direct consequences, the outcomes for payables were equal to a reduction of EUR 953 million. Thus, the total direct fiscal impact between 2020 and 2021 was, therefore, EUR 789 million. However, excluding pension payments averted, the total direct fiscal impact was equal to EUR –164 million. The receivables and payables as a result of two years of the COVID-19 pandemic per cost category are shown below in Figure 4.

#### 3.2.2. Indirect Consequences

##### Long-Term Population Health Effects: The Example of Long-Covid

The total number of people estimated to be limited in their daily lives through Long-Covid was 42,434 and 112,907 for 2020 and 2021, respectively. Furthermore, the number of individuals to be severely limited was equal to 18,648 in 2020 and 47,988 in 2021. The population data per age group are shown in Appendix A, Table A5a,b ((a) (2020) and (b) (2021)). Exemplary of the impact of long-term morbidity, Appendix A, Table A6a,b ((a) (loss of tax revenue) and (b) (social benefits paid)) show the effect of different levels of morbidity for an individual within different age groups. The annual revenue losses as a result of lower income are shown in Appendix A, Table A6a.

##### Costs of Emergency Policies

The expenditure of emergency policies in response to the COVID-19 pandemic in the Netherlands accounted for EUR 29 billion in the year 2020 and EUR 40 billion in 2021. These for the most part entailed liquidity measures for the private sector, subsidies and costs of emergency healthcare capacity. The ten largest expenses of emergency policies are shown in Appendix A, Table A7.

### 3.3. Approach II

#### 3.3.1. Change in Government Tax Income

**Figure 5:**
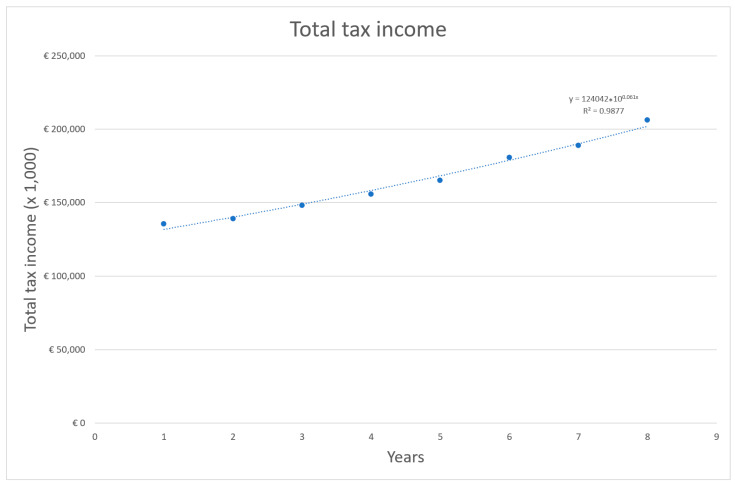
Total tax revenue over time between 2012 and 2019 [21].

Using an exponential function for extrapolation, the expected tax income values for 2020 and 2021 were equal to EUR 214,781 million and EUR 228,291 million. Comparing these expected values to the realized values for 2020 and 2021, respectively, EUR 205,671 million and EUR 223,819 million, showed a difference of EUR −9110 million and EUR −4472 million. The largest difference between expected and realized value before 2020 was in 2019 (EUR 4248 million) and in 2016 (EUR −3239 million) The total tax income over time between 2012 and 2019 is shown in Figure 5.

#### 3.3.2. GDP Opportunity Costs

**Figure 6:**
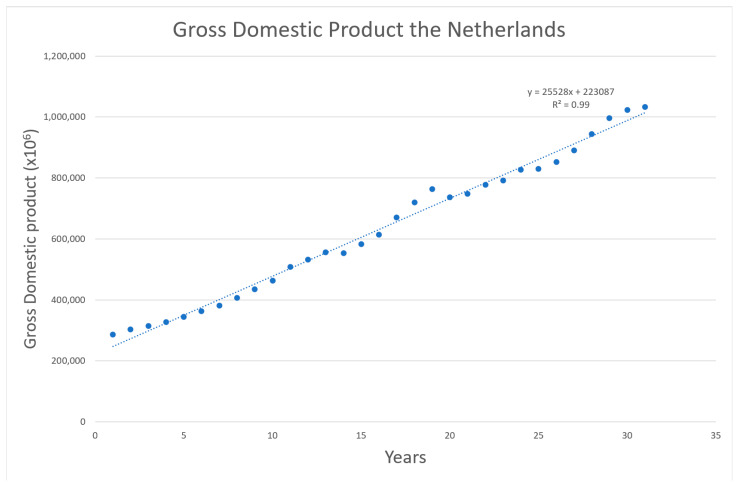
Dutch GDP development over time between 1990 and 2019 [39].

The fitted linear equation is used to calculate the expected values of 2020 equal to EUR 1.130 trillion compared to the realized value of EUR 1.034 trillion. This results in a difference between these two of EUR −96.296 billion. This was the largest difference observed, except for 2012. Comparable differences were seen in 2017. The mentioned data points, as well as the fitted function, are shown in Figure 6.

## 4. Discussion

The societal consequences of a CD outbreak are extensive. The disease itself, as well as potential emergency measures, affect every aspect of society, the impact of which is difficult to fully capture. However, the COVID-19 pandemic can be a learning case for future (hopefully smaller) CD outbreaks, as it highlights areas where society and government were affected the most. This framework—to our knowledge—is the first that utilizes these insights to allow analysis in the future for a more structured categorization of the specific impact areas of future outbreaks and policy interventions. By proposing two alternative approaches to the quantification of the fiscal impact, it is an intuitive orientation for researchers to analyse and communicate certain and uncertain aspects of the fiscal impact to decision-makers, while keeping the framework adaptable to specific disease contexts.

The approaches differ, however, in their value for analysing CD impact and policy interventions. Approach I, using detailed consequence data or assumptions, allows for prospective modelling of population data and thus a fiscal impact beyond the timeframe of the outbreak itself, yet might be restricted in its accuracy due to the limited information on impact categories before an outbreak occurred. On the other hand, Approach II, based on historical observations, can include all dynamics on state receivables and payables but can only be implemented retrospectively and limited to a specified timeframe because of its need for broad reported data. When comparing Approach I to Approach II, we see that the impact is much lower because this is only based on the disease data and the expected effects of this. Because Approach II assessed the total fiscal impact based on the expected and observed tax income, the observed losses are much higher than those calculated for the predetermined categories in Approach I. The difference between the two approaches is likely governed by the interaction of economic domains that give rise to multiplier effects. This is also consistent with previous observations noting that micro and macro models do not always corroborate [44]. Even though Approach II only assesses the fiscal impact in 2020 and 2021, while the impact of the COVID-19 pandemic is included in this, this also captures potential unrelated events or interventions.

Prospective disease modelling exercises used in health technology assessment together with an FHM analysis approach, as shown in Approach I, can be implemented to estimate the net value of that intervention in an already existing health economic methodology as part of an additional perspective. Through this approach, one can prospectively compare the damage averted and the needed investment by the state, especially relevant for decision-makers. FHM is an addition to the standard of HTA, valuing health units such as life-years or quality-adjusted life-years (QALYs) compared to costs [6]. Which approach to use is dependent on the data availability and goal of the analysis. If data are available, we recommend using both approaches in order to fully capture the fiscal impact. The FHM analysis simplifies the trans-sectoral comparison of government projects for general budgetary professionals when comparing funding needs from different ministries. Consequently, the benefit of the prevention of outbreaks could be more easily communicated.

Although COVID-19 has gained a significant amount of public attention, other CDs also affect daily life, especially that of individuals in vulnerable subpopulations. Children and the elderly affected by rotavirus, herpes zoster or influenza are well-known examples, yet societies oftentimes struggle to prevent these outbreaks. This is also partly because vaccination efforts within those populations have been deemed not to be cost-effective interventions; however, they may be justifiable on fiscal grounds. Regarding the fiscal impact, the direct consequences are expected to be minor for those groups as they are not part of the labour force. However, the indirect consequences could be notable, because their health does have repercussive effects on friends and family members who offer informal care or other support activities [45]. These dynamics could contribute to a sum of opportunity costs caused by a CD outbreak, which is not to be underestimated.

In addition, the fiscal impact of CD outbreaks, as shown in this study, and the response of governments to the COVID-19 pandemic could affect both the economic as well as the physiological implications of a disease outbreak [46]. Imposing a strict lockdown or alternating closures and reopening potentially creates a feeling of uncertainty for the population regarding economic prospects, financial responses of the government and recovery from the pandemic [46]. This could potentially affect the level of anxiety within a population as well as the economics. A more long-term strategic response to the COVID-19 pandemic, focused on a lower level of uncertainty, could potentially reduce the physiological and economic impact while restrictions remained longer [46]. The study by Busetta et al. concluded that better economic expectancies are associated with lower levels of anxiety [46]. Implementing FHM and improving the response, and therefore economic expectancies, could also substantially impact the population’s physiological state in addition to general health and governmental economic accounts. In a literature review by Della Monica et al. the pandemic had a substantial impact on population well-being due to depression, stress and fear of contagion; however, another literature reviews showed that this impact was minimal [47,48]. Comparing the fiscal impact of the CD outbreak, or pandemic, to the population health implications, including psychological implications, is key to understanding decision-making regarding response or preparedness.

Future research should focus on quantifying the presented framework for other CDs and countries. Transferability of consequence categories should be the main priority, as countries do have different designs of tax and social welfare systems. In this context, researchers should be careful to not use double-count costs, especially in the indirect effect areas. Lastly, future research should consider the prospective quantification of an outbreak by linking the epidemiological outcomes of a disease modelling exercise to the framework.

## 5. Conclusions

The impact of the COVID-19 pandemic has shown that better preparation and response in the Netherlands could potentially save millions. The FHM framework presented in this study is the first that proposes two fiscal approaches to quantify the fiscal impact of a CD outbreak to inform decision-making regarding prevention, public health and social services. The choice of approach is dependent on the goal as well as available data. Prospective analysis can be used in future investment decisions and health economic analysis, while retrospective identification is a valuable addition in the assessment of historic pandemics or CD outbreaks. Using the example case of COVID-19 in the Netherlands, it was shown that the cross-sectorial is extensive and can go far beyond only the fiscal consequences of infected and/or deceased individuals. The substantial fiscal impact as a result of mortality shows that investing in prevention can save substantial costs. While not all CDs would become a pandemic, a more proactive position from the government is warranted based on the potential fiscal impact.

The FHM framework for the Netherlands, as proposed in this manuscript, can be used by decision-makers and (health) economists to assess the fiscal impact and value of investing in prevention and/or interventions in health. The framework as presented in this manuscript could be translated to other disease areas or population data in order the calculate the fiscal impact. This could lead to a better substantiated investment decision and response to CD outbreaks. For society, this could mean that investments in public health, such as pandemic preparedness and prevention, have more value than previously acknowledged in economic analysis.

## 6. Strengths and Limitations

Our study adds a method for the assessment of health and policy interventions in the Netherlands. This creates an opportunity for decision-makers to invest in pandemic preparedness, public health and prevention. While we focused on the Dutch settings, some interventions might be better assessed at an international level, such as the European procurement of vaccines [49]. We showed the different inputs and methods for this assessment, and these methods can potentially be translated to other countries or an international level. However, more research is needed to prospectively communicate the value of interventions counteracting the severity and spread of CD as a clear-cut investment case to budgetary decision-makers. The limitation of Approach I is that it is dependent on the choice of categories to include as part of the analysis and the availability and validity of population data. A misrepresentation of infections, hospitalizations or mortality would result in an under- or over-estimation of the fiscal impact. The effects seen in the analysis are primarily the result of premature mortality; an under- or over-estimation of this would proportionately affect the outcomes. A limitation of Approach II is that the methodology includes all costs within a certain predetermined category, both related and unrelated to the disease. We recognize that this study has its limitations through its scoping literature review and inclusion of fiscal categories. The framework as proposed here can be supplemented and extended into other fiscal areas or detail. The key fiscal categories and primary contributors to fiscal impact are expected to be included in this manuscript.

## Figures and Tables

**Table 1:** Overview of fiscal categories identified through literature search and expert opinion.

Fiscal Category	Explanation	Adaptation	Source
Income tax	Income tax per age cohort, tax credit and labour participation.	Calculated weighted average per age cohort.	[15,16,17,18,27]
VAT	VAT per age cohort. No data were available for the 0–19 age cohort.	Average VAT percentage multiplied by the spendable income per age cohort.	[26,28]
Inheritance tax	Average inheritance tax. Not age specific.		[19,20,21,29]
Social benefits	Social benefits related to the inability to work. Fully or partly being unable to work.	Average social benefits per age cohort for the working population and different levels of inability to work are included.	[22,23,25,30,31,32]
State pension payments	State pension payment.	State pension per capita is calculated based on the total payments divided by people receiving state pensions.	[33]

## Data Availability

All data are available through their respective sources.

## Appendix A

**Table A1:** Average annual tax income per capita per age cohort (2020, NL).

0–19	-	EUR /year
20–39	4230	EUR /year
40–59	9370	EUR /year
60–79	3010	EUR /year
80+	1190	EUR /year

**Table A2:** VAT revenue per capita per age cohort.

0–19	-	EUR/year
20–39	2163	EUR/year
40–59	2833	EUR/year
60–79	2709	EUR/year
80+	2123	EUR/year

**Table A3:** Calculated percentage of Long-Covid per age group and severity.

Age Group	Activity Not Limited	Activity Limited	Activity Limited Severely
0–19	3.38%	2.16%	0.63%
20–39	4.38%	5.22%	1.98%
40–59	5.10%	6.83%	3.25%
60–80	3.18%	4.82%	2.42%
80+	1.85%	3.20%	1.63%

**Table A4:** (a) Population counts per age group and category based on raw data from the Dutch public health institute (RIVM) in 2020 (COVID-19 Dataset, 2022) [34]. (b) Population counts per age group and category based on raw data from the Dutch public health institute (RIVM) in 2021 (COVID-19 Dataset, 2022) [34].

(a)					
**2020**					
**Age Group**	**# of Infections**	**Hospitalisations**	**ICU Admissions**	**Mortality**	**Total**
0–19	114,134	354	10	0	114,498
20–39	261,868	1693	225	0	263,786
40–59	268,027	8170	1788	317	278,302
60–80	134,122	17,703	4356	4829	161,010
80+	47,935	8000	315	9956	66,206
Total	826,086	35,920	6694	15,102	
(b)					
**2021**					
**Age Group**	**# of Infections**	**Hospitalisations**	**ICU Admissions**	**Mortality**	**Total**
0–19	576,781	931	21	0	577,733
20–39	777,106	4215	685	0	782,006
40–59	629,319	13,584	3142	252	646,297
60–80	305,521	24,148	5682	3032	338,383
80+	68,206	9800	275	6209	84,490
Total	2,356,933	52,678	9805	9493	

**Table A5:** (a) Estimated long-Covid population numbers per age group. (b) Estimated Long-Covid population numbers per age group.

(a)			
	**2020**		
**Age Group**	**Activity Not Limited**	**Activity Limited**	**Activity Limited Severely**
0–19	3860	2468	718
20–39	11,478	13,666	5182
40–59	13,671	18,302	8716
60–80	4266	6465	3252
80+	888	1533	780
Sum			18,648
(b)			
	**2021**		
**Age Group**	**Activity Not Limited**	**Activity Limited**	**Activity Limited Severely**
0–19	19,505	12,470	3628
20–39	34,060	40,556	15,378
40–59	32,099	42,973	20,464
60–80	9718	14,726	7408
80+	1263	2182	1110
			47,988

**Table A6:** (a) Estimated loss of tax revenue due to morbidity per individual per year for different levels of work disability. (b) Added social benefits to be paid due to different levels of morbidity per individual per month.

(a)				
	**EUR /Year**	**EUR /Year**	**EUR /Year**	**EUR /Year**
	**25% Unable to Work (92.5% of Income)**	**50% Unable to Work (85% of Income)**	**75% Unable to Work (77.5% of Income)**	**Fully Unable to Work (70% of Income)**
0–19	-	-	-	-
20–39	317	634	952	1269
40–59	703	1405	2108	2811
60–79 (only for those working)	226	452	678	904
80+	-	-	-	-
(b)				
	**EUR /Month**	**EUR /Month**	**EUR /Month**	**EUR /Month**
	**25% Less Income from Work**	**50% Less Income from Work**	**75% Less Income from Work**	**Fully Unable to Work 3rd Month onwards (1st and 2nd Month)**
0–19 (>18 year)	273	546	819	1093
20–39	405 (434)	809 (868)	1215 (1301)	1619 (1734)
40–59	593 (635)	1187 (1271)	1779 (1907)	2373 (2543)
60–79 (only for those who work)	440 (471)	879 (942)	1319 (1413)	1758 (1884)
80+	-	-	-	-

**Table A7:** Expenditures per year and policy.

Emergency Policy	2020	2021
Emergency measure for retaining employment (NOW)	EUR 13,183,600,000	EUR 10,114,115,000
Corporate Rent/Utilities subsidy	EUR 1,069,000,000	EUR 6,486,000,000
Education disadvantage measures	EUR -	EUR 3,168,770,000
Testing capacity	EUR 949,000,000	EUR 3,524,000,000
Public Health agencies	EUR 387,000,000	EUR 2,922,000,000
Corporate Liquidity support (Tozo)	EUR 3,200,000,000	EUR 1,128,870,000
Development of vaccines, implementation and medication	EUR 93,000,000	EUR 1,754,000,000
Bonus for Healthcare workers	EUR 2,054,000,000	EUR 928,000,000
Subsidy for public transport	EUR 996,762,000	EUR 1,348,238,000
Purchase and distribution of medical goods	EUR 1,229,000,000	EUR 3,000,000
